# Biomass and nitrogen fixation dataset of *Pisum sativum* L. and *Vicia faba* L. cultivated under elevated CO_2_ and nitrogen addition

**DOI:** 10.1016/j.dib.2024.110644

**Published:** 2024-06-17

**Authors:** Ricardo A. Cabeza, Ricardo Pérez-Díaz, Ramón Amigo, Sebastian Salinas-Roco, Amanda Morales-González, Alejandro del Pozo

**Affiliations:** a Laboratory of Plant Nutrition, Department of Crop Sciences, Faculty of Agricultural Sciences, University of Talca, Talca, Chile; b Centro de Estudios en Alimentos Procesados (CEAP), Talca 3460000, Chile; c Plant Phenomics Center, Faculty of Agricultural Sciences, University of Talca, Talca, Chile

**Keywords:** Legumes, N_2_-fixation, Nodule density, Plant dry matter, Non-model legumes

## Abstract

It is expected that CO_2_ concentration will increase in the air, thereby stimulating the photosynthesis process and, hence, plant biomass production. In the case of legumes, increased biomass due to higher CO_2_ concentration can stimulate atmospheric nitrogen (N_2_) fixation in the nodules. However, N_2_ fixation is inhibited by external N supply. Thus, biomass production and N_2_ fixation were analysed in two legumes (*Pisum sativum* L. and *Vicia faba* L.) grown at two levels of CO_2_ and three N levels. *P. sativum* reduces fixation with high soil N (facultative), while *V. faba* maintains high fixation regardless of soil N levels (obligate). The N_2_ fixation and plant and nodule biomass of the two species were evaluated in a pot experiment under controlled conditions using growth chambers with artificial CO_2_ supply and N addition. The proportion of N derived from the air (%Ndfa) present in the plants’ biomass was calculated from the natural abundance of ^15^N and the N concentration of plant tissues using nonlegumes reference plants. Additionally, N content data are presented for both species growing at two levels of air CO_2_. The data may be useful for plant physiologists, especially those working on biological N_2_ fixation with non-model legumes at elevated CO_2_.

Specifications Table

**Table:** 

Subject	Plant Sciences, Plant physiology.
Specific subject area	Biological Nitrogen Fixation (BNF) of Legumes.
Type of data	Raw data in spreadsheets and graphs.
Data collection	Data were obtained from a controlled experiment in growth chambers with artificial addition of CO_2_ and N. The proportion of N derived from air (%Ndfa) was obtained by measuring the natural abundance of ^15^N and the N concentration of plant tissues with an elemental analyser connected to an isotope ratio mass spectrometer. Shoot, root and nodule biomass was determined by weighing and the N content was calculated based on N concentration and plant biomass.
Data source location	Laboratory of Plant Nutrition, Faculty of Agricultural Sciences, University of Talca, Talca, Chile.
Data accessibility	Repository name: Mendeley Data Data identification number: doi: 10.17632/tkz5zdjmz8.1 Direct URL to data: https://data.mendeley.com/datasets/tkz5zdjmz8/1 Instructions for accessing these data: The title of the data set is ‘Dry matter yield and nitrogen fixation of *Pisum sativum* and *Vicia faba* at elevated CO_2_ and three nitrogen levels`, the readers can access the dataset by typing in a browser the URL address: https://data.mendeley.com/datasets/tkz5zdjmz8/1

## 1 Value of the Data

• The dataset presented here is based on an experiment with two legumes, *Pisum sativum* L. and *Vicia faba* L., grown at ambient (*a*CO_2_) and elevated (*e*CO_2_) CO_2_ concentration and three N levels. The experiment was conducted under controlled conditions, and the biomass produced as well as the proportion of N derived from air (%Ndfa) were determined.
• The data set presents detailed information about the effect of artificial *e*CO_2_ on plant growth and on the%Ndfa when the N_2_ process is inhibited by N addition.
• This data set could aid in developing new legume varieties that are more efficient at N_2_ fixation and less susceptible to the N addition. Achieving this may involve traditional breeding and molecular biology research.
• The dataset may be useful to plant physiologists working with biological N_2_ fixation under climate change conditions with non-model legumes and can be used for modelling the N_2_ fixation process under different scenarios.

## 2 Background

Air CO_2_ concentration has increased due to the burning of fossil fuels, and it is expected to continue rising. In this context, the increment of ambient CO_2_ has been shown to stimulate photosynthesis and grain yield in C3 plants [1,2], while also provoking a dilution in the shoot N content of nonlegume crops [3,4], most likely due to limited soil N [5,6]. Studies conducted in Free-air CO_2_ enrichment (FACE) systems have shown that the yield increase under elevated CO_2_ (*e*CO_2_) was higher in legumes than in C3 cereals [2]. The higher productivity of C3 plants under *e*CO_2_ concentrations compared to plants grown at ambient CO_2_ (*a*CO_2_) has proven to trigger a high N demand, which in the case of legumes has resulted in an increment of the biological nitrogen fixation (BNF) [[7], [8], [9]]. In the case of soybean grown at *e*CO_2_, the C to N ratio has been found to be similar to that of plants grown at *a*CO_2_ [10], which could be possible because legume shoots can deliver more C to the nodules in order to fuel the BNF process [1,11]. This is likely achieved by either producing more nodules or increasing nodule size [12,13]. On the other hand, soil nitrogen (N) uptake by roots can inhibit the BNF process due to the lower energy costs that represent N absorption for the plant compared to the N_2_ fixation in the nodules [14].

There is evidence that the inhibition of N_2_ fixation in legumes varies by species, which can be classified as either facultative or obligate based on their response to soil N. *Pisum sativum*, for instance, is a facultative species because it reduces N_2_ fixation when soil N concentration is high. In contrast, *Vicia faba* is an obligate species, maintaining a high rate of N_2_ fixation regardless of soil N levels [15,16]. However, *e*CO_2_ increases the allocation of C to nodules, which can reduce the sensitivity of N_2_ fixation caused by N addition [17]. Therefore, elevated CO_2_ can help stimulate BNF and reduce its inhibition induced by soil N. The present dataset provides detailed information on the effect of elevated CO_2_ and N addition on biomass production and N_2_ fixation of two legumes: *Pisum sativum* L. and *Vicia faba* L. cultivated under controlled conditions in growth chambers with artificial CO_2_ addition.

## 3 Data Description

The dataset is composed of raw data of plant biomass production (shoot, root and nodules), nitrogen (N) concentration and natural abundance of isotopic N (^15^N) of two legume species (*P. sativum* L. and *V. faba* L.) grown at ambient and elevated CO_2_ (*a*CO_2_ and *e*CO_2_, respectively), and N addition. Furthermore, the dataset contains derived parameters from the raw data as root to shoot ratio (R/S), nodule density, the proportion of N derived from air (%Ndfa) and N content. To obtain the %Ndfa of legumes, references plants were cultivated at *a*CO_2_ and *e*CO_2_. The experiment was carried out at the Laboratory of Plant Nutrition, Faculty of Agricultural Sciences, University of Talca, Talca, Chile. The dataset is contained in a Microsoft® Excel® file with two sheets. The first sheet, ‘Dry matter`, contains the biomass produced under the above-mentioned conditions for *P. sativum* and *V. faba*.

**Fig. 1:**
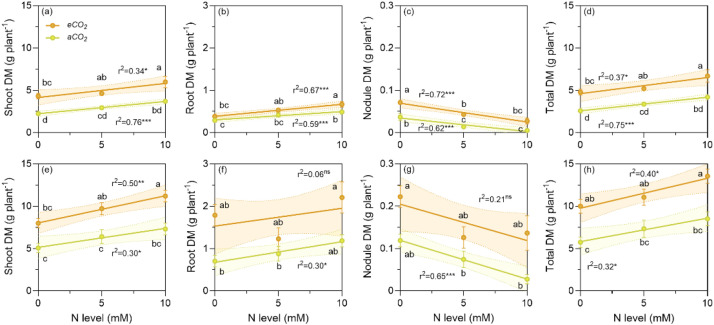
Shoot, root, nodule and total dry matter per plant of *P. sativum* (a to d) and *V. faba* (e to h) at elevated (*e*CO_2_= 1000 ppm) and ambient (*a*CO_2_= 400 ppm) CO_2_ and three nitrogen (N) levels. The increase in ambient CO_2_ led to an increase in shoot, root, and nodule biomass in both species due to an increment of C allocation, which in turn raised the N demand. Consequently, *e*CO_2_ level promotes N_2_ fixation. The N levels were zero (natural amount of available N in the soil/sand mix), 5 and 10 m M added as NH_4_NO_3_. The symbols represent the mean and the bars the standard error (*n* = 5 for each N level, i.e., *n* = 15 for each CO_2_ level, except for roots of *V. faba* at *a*CO_2_ with four replicates at N zero and for dry matter of nodules of *V. faba* at *a*CO_2_ with 5 and 10 mM of N where four replicates were recorded, respectively). r^2^ is the determination coefficient for the linear regressions, asterisks show the statistical significance at *p* values *<0.05, **<0.001 and ***<0.0001, respectively (ns= not significant). Small letters indicate differences among treatments according to two-way ANOVA and Tukey test (*p* < 0.05).

**Fig. 2:**
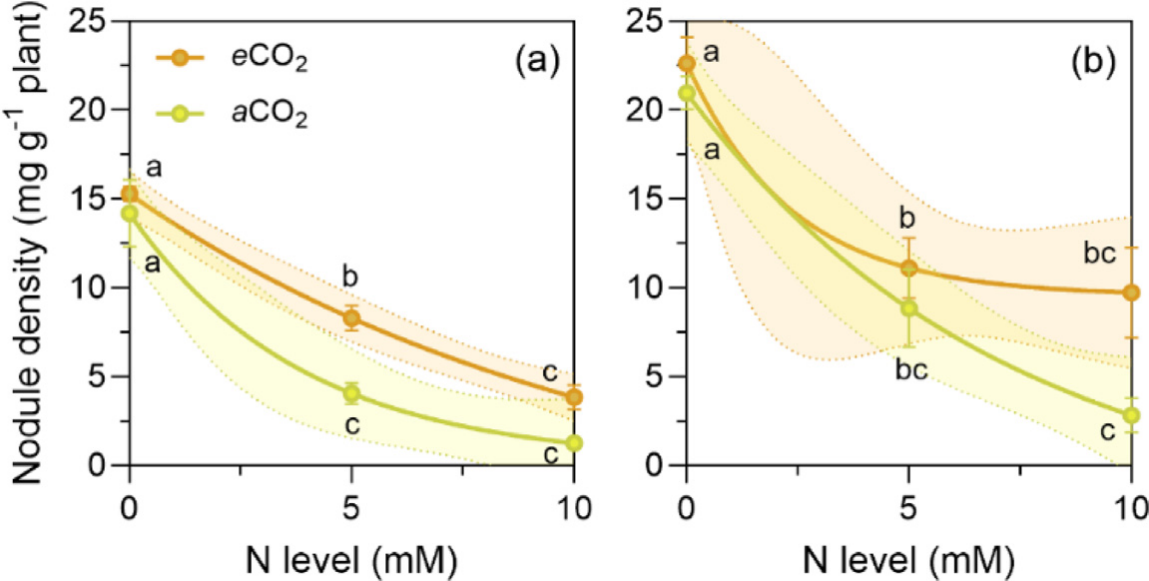
Nodule density of *P. sativum* (a) and *V. faba* (b) at elevated (*e*CO_2_= 1000 ppm) and ambient (*a*CO_2_= 400 ppm) CO_2_ and three nitrogen (N) levels. *e*CO_2_ led to a higher nodule density (mg g^-1^ plant), resulting in more N fixed per unit of dry matter produced compared to *a*CO_2_, particularly in *V. faba* (b), which can help to circumvent the negative effect of N addition. The N levels were zero, (the natural amount of available N in the soil/sand mix), and 5 and 10 mM were added as NH_4_NO_3_. The symbols represent the mean and the bars the standard error (*n* = 5 for each N level, i.e., *n* = 15 for each CO_2_ level, except *V. faba* at *a*CO_2_ with four replicates at 5 and 10 mM of N, respectively). r^2^ is the determination coefficient for the linear regressions, asterisks show the statistical significance at *p* values *<0.05, **<0.001 and ***<0.0001, respectively (ns= not significant). Small letters indicate differences among treatments according to two-way ANOVA and Tukey test (*p* < 0.05).

The data for dry matter of shoots, roots and nodules are composed of five replicates (Fig. 1), except for roots of *V. faba* grown at *a*CO_2_ where four replicates were recorded at N zero. Furthermore, the sheet ‘Dry matter` contains the derived parameters: R/S ratio, nodule density per plant and per root (Fig. 2). The data for nodule density are composed of five replicates, except for *V. faba* at *a*CO_2_ where four replicates were recorded at 5 and 10 mM of N, respectively.

**Fig. 3:**
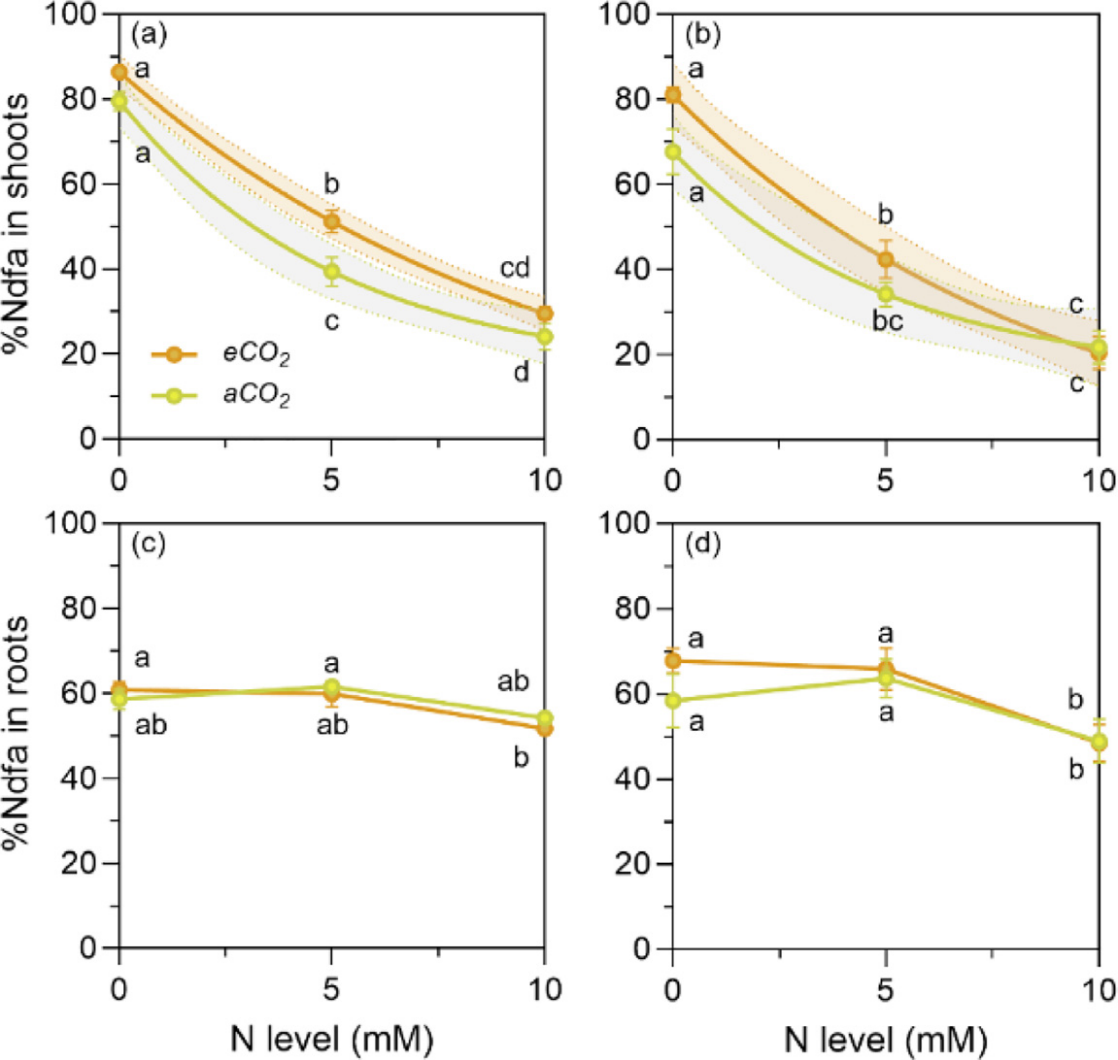
Proportion of N derived from air (%Ndfa) in shoots and roots of *P. sativum* (a and c) and *V. faba* (b to d) at elevated (*e*CO_2_= 1000 ppm) and ambient (*a*CO_2_= 400 ppm) CO_2_ and three nitrogen (N) levels. *e*CO_2_ did not affect significantly the %Ndfa (N_2_ fixation) and, although it promoted the nodulation, it did not prevent the inhibition of N_2_ fixation caused by N addition. The N levels were zero, (the natural amount of available N in the soil/sand mix), and 5 and 10 mM were added as NH_4_NO_3_. The symbols represent the mean and the bars the standard error (*n* = 5 for each N level, i.e., *n* = 15 for each CO_2_ level, except for roots of *V. faba* at *a*CO_2_ with four replicates at N zero). r^2^ is the determination coefficient for the linear regressions, asterisks show the statistical significance at *p* values *<0.05, **<0.001 and ***<0.0001, respectively (ns= not significant). Small letters indicate differences among treatments according to two-way ANOVA and Tukey test (*p* < 0.05).

**Fig. 4:**
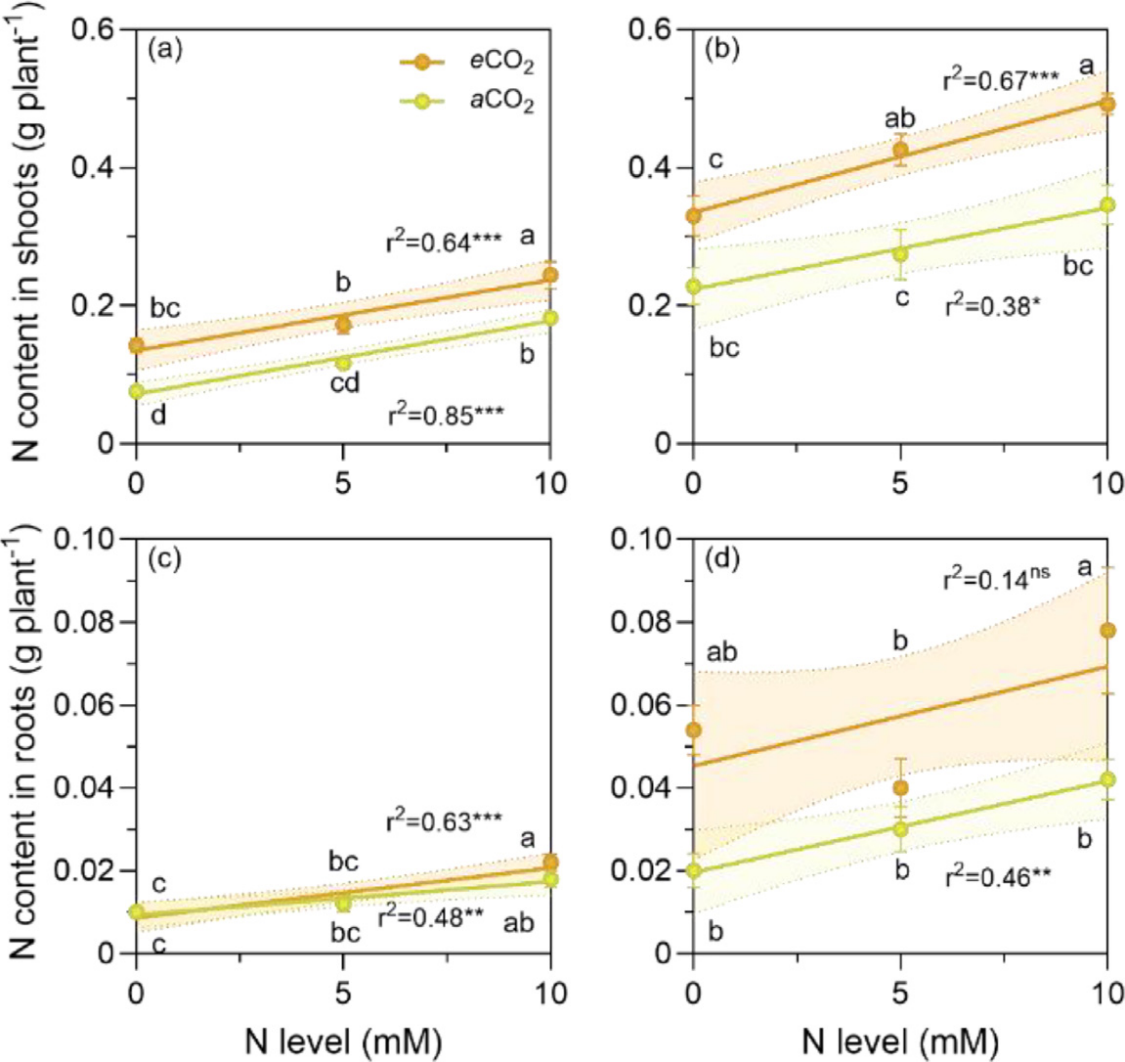
Nitrogen (N) content of shoots and roots of *P. sativum* (a and c) and *V. faba* (b to d) at elevated (*e*CO_2_= 1000 ppm) and ambient (*a*CO_2_= 400 ppm) CO_2_ and three nitrogen (N) levels. *e*CO_2_ resulted in higher N content compared to *a*CO_2_, particularly in the shoots of both species and in the roots of *V. faba*. This was due to increased biomass production and to the increase of N availability. Consequently, *e*CO_2_ promotes N uptake which could eventually prevent N leaching from the soil. The N levels were zero, the natural amount of available N in the soil/sand mix, and 5 and 10 mM were added as NH_4_NO_3_. The symbols represent the mean and the bars the standard error (*n* = 5 for each N level, i.e., *n* = 15 for each CO_2_ level, except for roots of *V. faba* at *a*CO_2_ with four replicates at N zero). r^2^ is the determination coefficient for the linear regressions, asterisks show the statistical significance at *p* values *<0.05, **<0.001 and ***<0.0001, respectively (ns= not significant). Small letters indicate differences among treatments according to two-way ANOVA and Tukey test (*p* < 0.05).

The next sheet ‘N-^15^N and %Ndfa` contains N concentration ([N] m/m%) and the natural abundance of ^15^N (δ^15^N ‰ vs Air) of shoots and roots of *P. sativum* and *V. faba* and of shoots for the following reference plants: wheat [*Triticum aestivum* L.], oat [*Avena sativa* L.], quinoa [*Chenopodium quinoa* Willd.] and sunflower [*Helianthus annuus* L.]. After the column with the natural abundance of ^15^N information, there are four columns with the values of %Ndfa calculated with each reference plant. The heads of the columns were abbreviated as %Ndfa (*Ta*), %Ndfa (*As*), %Ndfa (*Cq*) and %Ndfa (*Ha*), indicating the scientific names of wheat, oat, quinoa and sunflower, respectively. Then, the individual values of %Ndfa obtained with each reference plant were averaged (column ‘%Ndfa Average`) to obtain a more representative value of the %Ndfa for the legumes grown at the different conditions. The data for the %Ndfa of shoots and roots consist of five replicates, except for the roots of *V. faba* grown at *a*CO_2_ conditions, where only four replicates were recorded at zero N (Fig. 3). The same sheet contains the N content (g plant^-1^) separated into shoots and roots (Fig. 4), each with five replicates. Furthermore, the same sheet contains the N content (g plant^-1^) in shoots and roots for both species derived from air.

## 4 Experimental Design, Materials and Methods

The effects of elevated (*e*CO_2_; 1000 ppm) and ambient (*a*CO_2_; 400 ppm) CO_2_ concentrations and N addition on the biomass production and on the N_2_ fixation of *P. sativum* and *V. faba* were evaluated through an experiment under controlled conditions using growth chambers (Pitec ® BIOREF-38 L), with a 16 h/8 h light/darkness photoperiod and 25 and 16 °C, respectively. Because of the limited space inside the chambers, 2-L plastic pots were used with 3 kg of soil-sand mixture (1:1). The soil used was an Inceptisol with a pH of 6.3 (pHw 1:2.5) and 1.0, 20 and 196 mg kg^-1^ of N, P and K, respectively, 1.2 % of soil organic matter (SOM), and 0.03 dS m^-1^ of electric conductivity. Soil P level was increased by adding 200 mg of P per kg of soil as KH_2_PO_4_.

Seeds of *P. sativum* L. var. `Utrillo’ and *V. faba* L. var. `Super Agua Dulce’ were germinated in plastic clamshell containers with sterilized vermiculite and irrigated with distilled water. After 3–6 days the seedlings were transplanted to the 2-L plastic pots containing the soil-sand mixture, at a rate of two and one per pot of *P. sativum* and *V. faba* seedlings, respectively. Subsequently, they were inoculated with 4 mL/pot of a stationary *Rhizobium leguminosarum* bv. viceae YEM culture (local strains 373–3007-Su303 for *P. sativum*; and strain 1400 for *V. faba*), with an approximate cell density of 10^9^ mL^-1^ to promote the nodulation. Plants were fertilized with a nutrient solution composed of 0.7 mM of K_2_SO_4_, 0.5 mM of MgSO_4_, 0.8 mM of CaCl_2_, 4.0 µM of H_3_BO_3_, 0.1 µM of Na_2_MoO_4_, 1.0 µM of ZnSO_4_, 2.0 µM of MnCl_2_, 0.2 µM of CoCl_2_, 1.0 µM of CuCl_2_ and 1.0 µM of FeNaEDTA. Plants were grown at three N levels applied as ammonium nitrate (NH_4_NO_3_): 0, 5 and 10 mM. The basal nutrient solution plus N was applied at a frequency of every 4 days, with each application consisting of a volume of 200 mL. The soil water content was maintained at a 75 % water-holding capacity.

To modify the CO_2_ concentration inside the growth chambers, the CO_2_ was monitored and controlled by gas sensors (CO_2_ analyser Q-S151, Qubit Systems) and flowmeters (Aalborg®). The CO_2_ concentration was measured every five minutes by pumping out air flow from the chambers and analysing it with CO_2_ sensors. For this purpose, a plastic pipe was used to connect the inside of the growth chamber with an air pump that transferred the air to a CO_2_ sensor. When the CO_2_ concentration decreased due to the plants’ photosynthetic activity, CO_2_ was automatically released into the chambers by activating electric solenoid valves that connected a CO_2_ bottle with the chamber through a plastic pipe until the set-up concentration was restored (1000 o 400 ppm, respectively). A data logger (LabPro, Vernier®) and a digital control unit (DCU, Vernier®) were used to control sensors, flowmeters, and valves by means of the software Logger Pro®.

### 4.1 Plant material sampling

Shoots, roots, and nodule dry matter (DM) were evaluated at the flat pod stage. *P. sativum* was harvested 60 days after sowing (DAS), while *V. faba* was harvested 80 DAS. Shoots were separated from the roots/nodules and dried in an oven at 65 °C for 48 h until a constant weight was reached. Roots were carefully separated from the soil, washed, and stored at −80 °C. Then, nodules were separated from the roots manually with metal tweezers. Subsequently, roots and nodules were dried at 65 °C for 48 h for DM determination. Nodule density was calculated on the plant biomass base or on the root base as: nodule DM/(shoot+root DM) or nodule/root DM, respectively. Shoot and root DM were finely milled and stored in paper bags for further chemical analyses for determining the N concentration and ^15^N natural abundance.

### 4.2 Determining the proportion of N derived from air (%Ndfa)

The N concentration (%) and ^15^N natural abundance (expressed as ‰ δ^15^N relative to the ^15^N composition of atmospheric N_2_) were determined using an elemental analyser connected to an isotope ratio mass spectrometer, at the Laboratory of Applied Chemistry and Physics, at the University of Ghent, Belgium. Briefly, ca. 50 µg of N was weighed (depending on the N concentration in samples) in a tin cup to be analysed using an Elemental Analyser - Isotope Ratio Mass Spectrometer system (EA IsoLink™ IRMS-System interfaced through a ConFlo IV [Universal Interface for Continuous Flow Isotope Ratio MS] to a DELTA™ Q Isotope Ratio Mass Spectrometer, Thermo Fisher Scientific, Bremen). The δ^15^N values were normalised based on the AIR scale, using the following reference materials for quality control: USGS90 (Millet flour 8.84 ± 0.17 ‰ vs air) and USGS91 (Rice flour: 1.78 ± 0.12 ‰ vs ‰ vs air), and IA-R001–2019 (Wheat flour 2.55 ± 0.22 ‰ vs air). Standard deviation of repeated measurement was 0.08 ‰.

The calculation of the %Ndfa is based on the slight difference that exists between the ^15^N abundance of atmospheric N_2_ (δ^15^N = 0 ‰) and the ^15^N present in the soil (δ^15^N generally > 0 ‰) [18]. Tus, the %Ndfa in the legumes is calculated by comparing the natural ^15^N abundance of the legume (δ^15^Nleg) with that of reference plants (δ^15^Nref), using the following equation [19]:

$$\begin{array}{c} \%Ndfa=100\times \left[\frac{\delta^{15}N_{ref}-\delta^{15}N_{leg}}{\delta^{15}N_{ref}-\beta }\right] \end{array}$$

where δ^15^Nref represents the natural abundance of ^15^N present in the reference plant that comes from soil, δ^15^Nleg is the natural abundance in the legume´s tissue and the β value represents the ^15^N abundance in legumes that rely solely on N_2_ fixation for growth [18]. In this equation, the greater the difference in natural ^15^N abundance between the reference plant and the legume, the higher the N_2_ fixation and closer the δ^15^Nleg is to β. The reference plants used in this experiment were wheat (*T. aestivum* L.), oat (*A. sativa* L.), sunflower (*H. annuus* L.) and quinoa (*C. quinoa* Willd.). These plants were grown under the same experimental conditions as the legumes but only with the addition of 5 mM of N. The shoots of reference plants were harvested, dried, weighed, and ground in the same way as described above. The reference plants were harvested manually at the phenological stage of anthesis for oat and wheat or at full flowering for quinoa and sunflower. For calculation of the %Ndfa, the β values -0.65 and -0.5 were used for *P. sativum* and *V. faba*, respectively, according to Unkovich et al. [18].

### 4.3 Statistics

The experiments were arranged in a completely randomized design with five replicates per treatment. The effect of CO_2_ and N was assessed by linear and non-linear regressions and two-way ANOVA. Graphics, regressions, and ANOVA were plotted and calculated by using GraphPad Prism® version 10. Data were organized by using Microsoft® Excel® spreadsheet.

## Limitations

The dataset described here may be useful to plant physiologists working with nitrogen fixation process in non-model legumes, especially those working with climate change and CO_2_ increase scenarios. However, it should be taken into consideration that the experiment was conducted in growth chambers and not in a natural environment with increased CO_2_ concentration. It should also be considered that a better estimation of the β value of legumes can be obtained by cultivating legumes on an N-free substrate. This approach ensures that all the N present in the plant tissue originates solely from the BNF process.

## Ethics Statement

The authors have read and followed the ethical requirements for publication in Data in Brief and confirm that the current work does not involve human subjects, animal experiments, or any data collected from social media platforms.

## Credit Author Statement

**Ricardo Pérez-Díaz, Ramón Amigo, Sebastian Salinas-Roco** and **Amanda Morales-González** performed most of the experiments; **Ricardo A. Cabeza** set up the CO_2_ control system; **Ricardo A. Cabeza** and **Alejandro del Pozo** organized the data presentation; conceived of the project and funding and wrote the article with contributions from all of the authors. All authors have read and agreed to the published version of the manuscript.

## Data Availability

Dry matter yield and nitrogen fixation of Pisum sativum and Vicia faba at elevated CO2 and three nitrogen levels (Original data) (Mendeley Data).
